# Strategic vaccination responses to Chikungunya outbreaks in Rome: Insights from a dynamic transmission model

**DOI:** 10.1371/journal.pntd.0012713

**Published:** 2024-12-09

**Authors:** Albertus Constantijn Sloof, Martijn Boer, Gerard T. Vondeling, Adrianne M. de Roo, Juan Carlos Jaramillo, Maarten J. Postma

**Affiliations:** 1 Department of Health Sciences, University Medical Center Groningen, Groningen, Netherlands; 2 Asc Academics B.V., Groningen, Netherlands; 3 Valneva Austria GmbH, Vienna, Austria; 4 Vaccines Europe, Executive Board Member, Brussels, Belgium; 5 Department of Economics, Econometrics and Finance, University of Groningen, Faculty of Economics & Business, Groningen, Netherlands; 6 Center of Excellence for Pharmaceutical Care Innovation, Universitas Padjadjaran, Bandung, Indonesia; 7 Division of Pharmacology and Therapy, Faculty of Medicine Universitas Airlangga, Surabaya, Indonesia; National University of Singapore Public Health, SINGAPORE

## Abstract

**Background:**

Chikungunya virus (CHIKV) outbreaks, driven by the expanding habitat of the *Aedes albopictus* mosquito and global climate change, pose a significant threat to public health. Our study evaluates the effectiveness of emergency vaccination using a dynamic disease transmission model for a potential large-scale outbreak in Rome, Italy.

**Methods:**

The model incorporates a susceptible-exposed-infected-recovered (SEIR) framework for human and mosquito populations, taking into account temperature-dependent mosquito lifecycle dynamics, human-mosquito interactions, and various vaccination scenarios.

**Findings:**

Results indicate that emergency vaccination could significantly mitigate the impact of a CHIKV outbreak. Without vaccination, an outbreak is estimated to infect up to 6.21% of Rome’s population, equating to approximately 170,762 individuals. Implementing rapid vaccination after detecting the virus in ten individuals and achieving 40% coverage could reduce infection rates by 82%, preventing 139,805 cases. Scenario and sensitivity analyses confirm that even with lower vaccination coverage rates, significant benefits are observed: at 10% coverage, the number of infections drops to 115,231, and at 20% coverage, it further reduces to 76,031. These scenarios indicate prevention of approximately 33% and 55% of infections, respectively.

**Conclusions:**

The findings highlight the critical role of timely vaccination interventions in outbreak settings, demonstrating that even modest coverage levels can markedly decrease the spread of CHIKV. This study underscores the importance of preparedness, early detection and adaptive response capabilities to manage emerging infectious diseases in urban centres, advocating for strategic vaccine stockpiling and rapid deployment mechanisms to enhance public health outcomes.

## Author summary

Chikungunya virus (CHIKV) outbreaks, exacerbated by the spread of the *Aedes albopictus* mosquito and climate change, are a growing public health concern. Our study explores the potential impact of emergency vaccination during a large-scale CHIKV outbreak in Rome, Italy, using a dynamic model that simulates disease transmission among humans and mosquitoes.

We found that emergency vaccination could significantly reduce the number of infections. Without vaccination, an estimated 6.21% of Rome’s population, about 170,762 people, could be infected. However, starting vaccination after detecting ten cases and reaching 40% coverage could decrease infections by 82%, preventing 139,805 cases. Even with lower coverage rates, the benefits are substantial: 10% coverage could prevent 33% of infections, and 20% coverage could prevent 55% (S2 Fig).

Our findings emphasize the importance of quick and effective vaccination strategies during outbreaks. Preparedness, early detection, and flexible responses are crucial for managing emerging infectious diseases in cities. This study supports the need for strategic vaccine stockpiling and rapid deployment to protect public health.

## Introduction

Chikungunya is a viral disease caused by the chikungunya virus (CHIKV) that is primarily transmitted by infected mosquitoes of the *Aedes aegypti* and *Aedes albopictus* species [1,2]. Chikungunya is a disease with a high morbidity rate as well as potentially debilitating long-term consequences and no specific treatments or preventive vaccines [3,4]. The name chikungunya originates from the Kimakonde language, meaning ‘to be contorted’, owing to the severe joint pain that is the main symptom both in the acute and chronic phases of the disease [4–7]. Between 43% (based on people with laboratory-confirmed or self-declared chikungunya) and 51% (based on people with laboratory-confirmed symptomatic chikungunya) of patients will develop chronic chikungunya, which is primarily characterized by arthralgia and/or fatigue that can last for months or even years [7,8]. The percentage of patients with chronic chikungunya decreases over time from 43.89% at three months, 34.39% at six months, and 31.87% at 12 months [9]. Due to the long-term disability and medical burden of the disease, the majority of those who experience chronic CHIKV infection do not return to their previous health status for multiple years ranging from approximately 70% after 1.5 years to 57% after 2.5 years [10,11].

During recent years the *Ae*. *albopictus* mosquito has begun to colonize Europe, having become more adapted to colder climates [12,13]. The first importation of *Ae*. *albopictus* in Europe was recorded in 1979 in Albania, where it became established. Italy was the second European country where *Ae*. *albopictus* was found, in Genoa in 1990, and is currently the most infested European country. Since 2000, *Ae*. *albopictus* also became established on the Cote d’Azur in southern France [12]. Moreover, due to global warming, it is expected that the habitat of the *Ae*. *albopictus* mosquito will expand further northwards [14]. The habitat expansion of *Ae*. *albopictus* and its effectiveness as a disease transmission vector for CHIKV has resulted in multiple small outbreaks in Europe, particularly in Italy and France [15]. The cold winter temperatures in Europe prevent a year-round presence of *Ae*. *albopictus*, possibly preventing CHIKV from becoming endemic. Therefore, outbreaks of CHIKV in Europe occur in summer and result from infected individuals travelling from an endemic region, such as Latin America, Africa or Asia. The risk of new CHIKV outbreaks in Europe is substantial due to a lack of immunity, a suitable climate and vector presence, and importation through infected travellers [16]. During summer the European climate and ecological conditions allow for autochthonous transmission of CHIKV, leading to potential outbreaks [17].

The first outbreak of CHIKV in Europe occurred in 2007 in Ravenna, Italy. The outbreak resulted in 200 reported cases and 10% of the population being exposed to the virus [18]. The most recent European CHIKV outbreak occurred in 2017 in Anzio, Italy, and resulted in 414 reported cases [19]. The outbreak resulted in two additional foci in Rome and Latina. The earliest symptom onset in Anzio was reported on the 26^th^ of June, and in Latina and Rome this occurred on the 13^th^ and 20^th^ of August, respectively. In Rome a total of 80 cases were recorded in the metropolitan area [20].

Despite the limited number of chikungunya cases identified in Rome in 2017, large urban centres are particularly vulnerable to larger outbreaks compared to rural areas [21], especially when introduction of the virus occurs at the time when conditions are optimal for disease spread. This underscores the importance of outbreak preparedness, especially in the light of globalization and climate change. In November 2023, IXCHIQ, the first chikungunya vaccine was approved by the FDA [22]. This vaccine presents a strategic tool to mitigate the impact of outbreaks by flattening the epidemic curve. The sterilizing immunity induced by IXCHIQ has been established through the surrogate immunogenicity endpoint based on non-human primate passive transfer studies using human sera corroborated by findings from a seroepidemiological study [23]. This surrogate endpoint—the seroresponse rate defined as virus neutralizing antibody titer ≥150 determined by μPRNT50—was developed and agreed with the health authorities (United States Food and Drug Administration, Health Canada, and the European Medicines Agency) which considered the endpoint to be acceptable to support licensure via the accelerated approval pathway [24–26]. In the pivotal phase 3 trial, IXCHIQ met its primary endpoint with 98.9% of vaccinated individuals reached the predefined level of neutralizing antibodies [27].

Vaccination strategies can be broadly categorized into routine vaccination programs, which inoculate a segment of the population as a preventative measure, and emergency vaccination programs, which are deployed reactively to curb the spread of an outbreak. Our modelling efforts are designed to assess the potential magnitude of a chikungunya outbreak in Rome and evaluate how effectively emergency vaccination can reduce the number of infections and thus the associated burden of disease from both acute and chronic chikungunya cases. By using modelling techniques, we aim to estimate the potential size of an outbreak of CHIKV in case it would originate in Rome and evaluate how effectively emergency vaccination can reduce the number of infections.

## Methods

**Fig 1:**
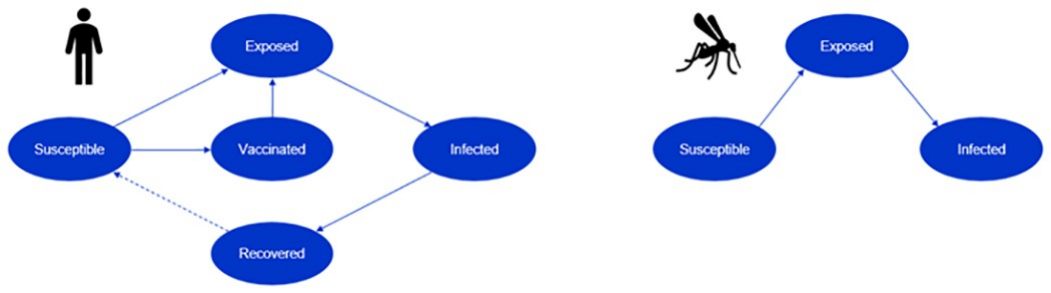
SEIR model structure for humans and mosquitoes. The development of CHIKV infection within the populations.

To investigate the potential size of an outbreak in Rome and the impact of emergency vaccination, a dynamic disease transmission model was created. The model includes both a mosquito population and a human population to be able to investigate the human-to-mosquito-to-human transmission of CHIKV. The disease transmission between the populations and infectivity within populations are modelled using a susceptible, exposed, infected, recovered (SEIR) model, see Fig 1. The size of the mosquito population is determined endogenously in the model using temperature-dependent egg-laying, development, and mortality rates. Due to the temperate climate in Italy, the mosquito population will die during winter with eggs surviving in a diapaused state. Therefore, the outbreak will not last for more than one year. A simplifying assumption was made that the human population is constant over the time horizon of one year. Therefore, no human births and deaths are included in the model.

### Mosquito population dynamics

The mosquito population in the model was divided into four developmental stages: eggs (E), larvae (L), pupae (P), and adults (A). The eggs can either enter the model in a diapaused state (E_D_) when temperatures are too low or as normal eggs in temperature ranges where hatching can occur. Furthermore, male adults are not taken into account as only female adults can carry CHIKV and transfer it to humans. The development of the lifecycle stages over time is described by the following set of differential equations, which has been adapted from an earlier published model [28]:

**System of Equations 1 Determining the Mosquito Population Dynamics**.

$$\begin{array}{l} {\dot{E}}_{D}\;=\;p_{D\;}n_{E}\frac{A}{L_{A}}exp\left(1-\frac{E}{C}\right)-m_{D}E_{D}-d_{D}E_{D}\\ {\dot{E}}_{E\;}=\;d_{D}E_{D}+\left(1-p_{D}\right)\;n_{E}\frac{A}{g}exp\left(1-\frac{E}{C}\right)-m_{E}E_{E}-d_{E}E_{E}\\ \dot{L\;}=\;d_{E}E_{E}-m_{L}L-d_{L}L\\ \dot{P}\;=\;d_{L}L-m_{P}P-d_{P}P\\ \dot{A}\;=\;p_{F}\;d_{P}\;P-m_{A}A \end{array}$$

Where *p*_*D*_ is the probability of an egg being laid in the diapaused state, *n*_*E*_ is the number of eggs per gonotrophic cycle, *L*_*A*_ is the gonotrophic cycle length, *C* is the egg carrying capacity, *p*_*F*_ is the proportion of adults that are female, *m* refers to mortality and *d* to development. The subscripts used refer to the relevant lifecycle development stages with *D* referring to diapause, *E* to non-diapaused eggs, *L* to larvae, *P* to pupae, and *A* to adults.

The development from early lifecycle stages to later ones and mortality rates per lifecycle stage is dependent on temperature. This system of equations was obtained from an earlier study of CHIKV in Italy [28]. The mortality rates are based on laboratory studies, with higher mortality likely occurring in an uncontrolled situation. Therefore, a multiplier of 1.35 was applied to early lifecycle mortality and a multiplier of 4 was applied to adult mortality following the earlier study of CHIKV in Italy [28]. The formulas for temperature-dependent development duration and mortality rates are described in Table 1 and Table 2. The inverse of the development duration was used for the development rates in System of Equations 1.

**Table 1:** Duration of development between lifecycle stage of mosquitoes [28].

Lifecycle stage		Formula	P_1_	P_2_	P_3_
Eggs	L_E_	$P_{1}-P_{2}*\text{exp}\left(-{\left(\frac{T-20}{P_{3}}\right)}^{2}\right)$	6.5	4.0	4.1
Larvae	L_L_	P_1_ * T^2^ + P_2_ * T + P_3_	0.12	-6.6	98
Pupae	L_P_	P_1_ * T^2^ + P_2_ * T + P_3_	0.027	-1.7	27.7
Gonotrophic cycle	L_A_	P_1_ * T^2^ + P_2_ * T + P_3_	0.046	-2.77	45.3

P, polynomial; T, temperature.

**Table 2:** Mortality rates per lifecycle stage [28].

Lifecycle stage		Formula	P_1_	P_2_	P_3_
Eggs	*M*_*E*_	$P_{1}-P_{2}*\text{exp}\left(-{\left(\frac{T-25}{P_{3}}\right)}^{6}\right)$	506.18	506	27.3
Larvae	*M*_*L*_	P_1_ + P_2_ * exp(T–P_3_)	0.029	858	43.4
Pupae	*M*_*P*_	P_1_ + P_2_ * exp(T–P_3_)	0.021	37	36.8
Adults	*M*_*A*_	P_1_ + P_2_ * exp(T–P_3_)	0.031	95820	50.4

P, polynomial; T, temperature.

For the temperature inputs, daily average local temperatures were used. The average was calculated as the unweighted average between the minimum and the maximum temperatures per day [29]. Polynomial functions were fitted to the daily average temperatures to perform smoothing of the temperatures over time, and a third-order polynomial was considered to provide the best fit. The raw temperature time series and the smoothed polynomial functions are depicted in S1 Fig. The other polynomials are included in the scenario analyses and assess the impact of different temperatures across years. Data smoothing was performed to reduce noise and remove outliers, thereby providing a more reliable representation of the temperature data.

The egg, larvae, and pupae mosquito stages are aquatic; therefore, a transformation was applied from daily air temperature to water temperature using.

**Equation 2. Transformation of Air Temperature to Water Temperature** [30].

$$T_{W}=\beta_{0}+\beta_{1}T_{A}$$

Where T_W_ represents the temperature of the water and T_A_ is the air temperature. β_0_ is equal to 5.08 and β_1_ to 0.752 [30]. The water temperature was used for the early lifecycle stages, while the adults are affected by the air temperature.

### Human-mosquito interaction

The spread of the chikungunya virus from human to mosquito to human are dependent on the interactions between humans and mosquitoes and the development of the infection within individuals from both species. The development of the infection determines the movement from susceptible, to exposed, infectious, and recovered over time. The interactions between humans and mosquitoes are regulated by the biting rate which is determined by the gonotrophic cycle length, the number of bites per cycle, and the percentage of human feeding. The formulae regulating the infection development for both species and the transmission between species are described in System of Equations 3 and Table 3.

***System of Equations 3 Determining the Infection Development and Transmission***.

$$\begin{array}{l} {\dot{H}}_{S}\;=\;-\frac{BP}{L_{A}}S_{H}A_{i}\frac{H_{S}}{H}-H_{S}\frac{V_{Cov}}{V_{Dur}}V_{T}\\ {\dot{H}}_{E}\;=\;\frac{BP}{L_{A}}S_{H}A_{i}\frac{H_{s}}{H}+\frac{BP}{L_{A}}S_{V}^{T}A_{i}\frac{H_{V}}{H}-I_{H}H_{E}\\ {\dot{H}}_{I}\;=\;I_{H}H_{E}-R_{H}H_{I}\\ {\dot{H}}_{R}\;=\;R_{H}H_{I}\\ {\dot{H}}_{V}\;=\;H_{S}\frac{V_{Cov}}{V_{Dur}}V_{T}-\frac{BP}{L_{A}}S_{V}^{T}A_{i}\frac{H_{V}}{H}\\ {\dot{A}}_{S}\;=\;\dot{A}-\frac{BP}{L_{A}}S_{A}A_{S}\frac{H_{I}}{H}\\ {\dot{A}}_{E}\;=\;\frac{BP}{L_{A}}S_{A}A_{S}\frac{H_{I}}{H}-I_{A}A_{E}-M_{A}A_{E}\\ {\dot{A}}_{I}\;=I_{A}A_{E}-M_{A}A_{I} \end{array}$$

**Table 3:** Interpretation of parameters determining infection development and transmission.

Parameter	Interpretation
H	Humans
B	Number of bites per mosquito per gonotrophic cycle
P	Percentage of bites targeting humans
L	Lifecycle stage, see Table 1
S	Susceptibility
A	Adult *Ae*. *albopictus* mosquito
V	Vaccination
I	Incubation rate
R	Recovery rate
M	Mortality
Å	Adult mosquito population dynamics, see System of Equations 1
**Superscripts and subscripts**	
S	Susceptible
H	Human
I	Infectious
Cov	Final coverage rate
Dur	Duration until final coverage rate in days
T	Time
E	Exposed
V	Vaccinated
R	Recovered
A	Adult *Ae*. *albopictus* mosquito

The transmission of CHIKV occurs when an infectious mosquito bites a susceptible human or when a susceptible mosquito bites an infectious human. Different rates of human and mosquito susceptibility were allowed, these rates represent the probability of the transfer of the chikungunya virus when a human is bitten by an infectious mosquito or when a susceptible mosquito bites an infectious human respectively. To determine the infectiousness of humans and mosquitoes the SEIR model presented in System of Equations 3 was used. Individuals move from susceptible to exposed when CHIKV is transmitted. The incubation rate determines the movement to the infected state, where they are able to transmit the disease. The model includes both an intrinsic and extrinsic incubation rate, where the former determines the percentage of humans in the exposed state that moves from exposed to infected per day and the latter determines the same movement for mosquitoes. The recovered state was included for individuals who have been infected with CHIKV and have gained natural immunity against reinfection. The recovery rate determines the percentage of individuals in the infectious state that moves to the recovered states per day. The incubation and recovery rates are shown in Table 4. Mosquitoes remain infected for their remaining life span.

**Table 4:** Overview of the parameters used in the model.

Parameter	Value	Source
**Human parameters**		
Date first exposure	05 June	Assumed
Human susceptibility	65%	[28]
Intrinsic incubation rate	33%	[37]
Recovery rate	17%	[6]
**Mosquito parameters**		
Mosquito susceptibility	77.5%	[38]
Extrinsic incubation rate	33.3%	[38]
Mortality multiplier aquatic stages	1.35	[28]
Mortality multiplier adults	4	[28]
Percentage female	50%	Assumed
**Biting rate parameters**		
Blood meals per gonotrophic cycle	1.5	Assumed
Percentage of human feeding in rural areas	22.7%	[32]
Percentage of human feeding in urban areas	95.7%	[32]
Rural population density	469.7 humans per km^2^	[34]
Urban population density	2250 humans per km^2^	Assumed
**Vaccination parameters**		
Cases until outbreak identification	10	Assumed
Delay from outbreak identification to vaccination rollout	14 days	Assumed
Duration of vaccine rollout	30 days	Assumed
Vaccine effectiveness for preventing infection	98.9%	[27]
Vaccine coverage	40.0%	[35]
Time to immunity post-vaccination	14 days	[27]

The human-mosquito interactions are assumed to follow a homogenous mixing model, which means that mosquitoes are equally likely to bite each human included in the model regardless of location, age, or other characteristics. It is recognized that the probability of being bitten by an infected mosquito is likely to depend on these characteristics; however, their inclusion would require additional assumptions with regard to human and mosquito travelling behaviour and age-dependent time spent outdoors. These assumptions would have been based on limited or no data, therefore the homogenous mixing model was used instead.

Mosquitoes require a blood meal to provide the nutrients needed for egg production [31]. *Ae*. *albopictus* can feed multiple times per gonotrophic cycle and can feed on multiple species, although in more urbanized areas they seem to have a preference for humans [32]. In the model, mosquitoes are assumed to feed one and a half times per gonotrophic cycle as *Ae*. *albopictus* take multiple bloodmeals during a gonotrophic cycle [33]. In addition, the percentage of the bloodmeals taken from humans increases with population density. We assumed the effect of population density to be linear based on human-feeding measurements taken from a farm outside Rome and another location near a university in the centre of Rome [32]. The percentage of mosquitoes that had fed on humans was measured, however the concurring population densities at the trap sites were not available. The population density for the farm outside Rome were obtained from census data [34], however population density for the area surrounding the university could not be obtained and thus was based on an assumption. Therefore, the biting rate depends on both temperature and population density. The values used for the development of the virus in human and mosquito population and the biting rate are shown in Table 4.

### Vaccination

Vaccination was modelled considering IXCHIQ, the first chikungunya vaccine approved by FDA and the EMA. The SEIR model was adapted to include an additional state, to account for vaccinated individuals. The vaccine induces sterilizing immunity, therefore it prevents the spread of the virus and provides indirect protection beyond those who are vaccinated. Vaccination is simulated as an emergency response vaccination programme, assuming a local stockpile of vaccines would be available for use in case of an outbreak. In line with the FDA and EMA approval, during the emergency vaccination programme all individuals over the age of 18 would be eligible for vaccination. The outbreak was assumed to be detected once ten individuals had recovered from the infection. The number of individuals in the recovered state were used instead of the number of infections, because individuals do not move out of the recovered state, simplifying the implementation. Furthermore, laboratory confirmation of an infection takes time meaning that an individual could have reached the recovered state prior to laboratory confirmation of their infection. Scenario analyses were performed to demonstrate the uncertainty and model sensitivity around this parameter.

Vaccine administration was assumed to start two weeks after outbreak identification with a one-month period to reach the maximum vaccine coverage rate, defined as the percentage of the entire population that has received the vaccination. No previous emergency vaccination programmes for a chikungunya outbreak have been implemented, therefore the maximum vaccination rate is difficult to estimate. During the COVID-19 emergency vaccination programme, 40% of the Italian population received the vaccination, which is assumed to be the upper limit of the vaccination coverage rate [35]. The impact of vaccination was investigated for maximum vaccination coverage rates of 10%, 20%, 30%, and 40% to demonstrate the uncertainty around this parameter. Since 15.38% of the population of Rome is younger than 18 years old [36], these vaccination coverage rates of the total population translate to 11.8%, 23.6%, 35.5% and 47.3% of the eligible population. In absolute numbers, these rates equate to vaccinating 274,903, 549,806, 824,709, and 1,099,612 individuals, respectively when considering to spread vaccination across the entire population. In case the most at-risk areas are targeted the absolute numbers could be reduced while maintaining the protective efficacy could be maintained. This possibility was not further evaluated in the current study. In individuals who have received the vaccination, it takes up to two weeks until the vaccine provides full protection against CHIKV [25]. No intermediate protection is assumed between vaccination and full protection after two weeks. Vaccine effectiveness is assumed to be 98.9%, based on immunogenicity data from the phase 3 clinical trial evidence of IXCHIQ [27]. The values used for the vaccination intervention and effectiveness are shown in Table 4.

### Calibration

To estimate the size of the outbreak in Rome, data from the most recent Italian chikungunya outbreak in Anzio were used to calibrate the model.

From the ecological model input parameters needed to describe the mosquito dynamics, the main unknown parameter is the egg-carrying capacity, which describes the number of eggs that can be sustained within an area. A larger egg-carrying capacity in an area allows for more mosquitoes to be born resulting in a larger mosquito population. A larger mosquito population makes an outbreak more likely and increases the predicted size of the outbreak. The egg-carrying capacity depends on the number of stagnant water sources, such as in puddles, buckets, or car tyres [39].

The model was calibrated to align with the Anzio outbreak through adjustment of the egg-carrying capacity parameter, thus influencing the size of the mosquito population. The parameters used for calibration are presented in Table 5. The egg-carrying capacity was adjusted until the number of people infected with chikungunya predicted by the model matched the number of cases reported for the Anzio outbreak. To allow the change of the setting of the outbreak from Anzio to Rome it was assumed that the egg-carrying capacity was proportional to land area. Given that the metropolitan area of Rome is larger than the Anzio regio, a larger egg-carrying capacity was required. The assumption that the egg-carrying capacity is proportional to land area is supported by similar numbers of *Ae*. *albopictus* that are found in traps in rural and urban areas surrounding Rome [32]. Furthermore, to prevent overestimation of the Rome outbreak it was assumed that in an urbanized area the number of breeding grounds might be slightly lower. Therefore, the estimated egg-carrying capacity for the Rome scenario was reduced by 5%.

**Table 5:** Parameters used for calibration to anzio and transfer to Rome.

Parameter	Anzio	Rome	Source
Human population size	54,311	2,749,031	[40]
Population density (humans per KM^2^)	1,342.2	2,135.6	[40]
Egg carrying capacity	137,500	3,851,253	Calibration and calculation
Initial number of eggs	75,000	2,100,684	Calibration and calculation
Land area (KM)	43.66	1287.24	[40]
Land area proportionality (%)		95%	Assumed
Vector-host ratio range	1.9–7.3		[41]

### Validation procedure

To validate the calibration, the obtained maximum vector-host ratios were calculated both for the Anzio and the Rome scenario and compared to reported vector-host ratios during the Anzio outbreak [41]. The vector-host ratio refers to the number of mosquitoes (vectors) that are present per human (host). Furthermore, the vector-host ratio for Rome needs to be lower than the ratio for Anzio as vector-host ratios tend to decrease with population density [42].

To validate the estimated outbreak size in Rome, a search for similar outbreaks was performed. Three types of outbreaks were considered to be similar to outbreaks in Rome: outbreaks with a similar vector-host ratio, outbreaks in Europe, and the focus in Rome of the Anzio outbreak. Outbreaks with a similar vector-host ratio were expected to result in similar outbreak sizes as the size of the mosquito population is relatively similar, and outbreaks in Europe were considered similar given similar geographic and climatic conditions.

These outbreaks were simply compared to the estimated outbreak for similarity in outbreak size and potential differences in circumstances that could explain differences in outbreak size. The focus of the Anzio outbreak that occurred in Rome occurred at a later date during the outbreak while in the estimated outbreak the start date of the Anzio outbreak is used. Changing the start date to the Rome outbreak should result in the same size as was reported for this focus.

### Sensitivity and scenario analyses

The benefit of any vaccination program is highly dependent on the vaccine coverage that will be achieved. As it is unknown how many people would comply to emergency vaccination for the disease, a scenario analysis was performed for the coverage rate. This rate was varied from 0% to 100% by increments of 10% to depict the number of infections over time graphically.

Many of the parameters related to the mosquito population and the transmission of CHIKV were reported in literature within a substantial range suggesting a large degree of uncertainty around these parameters. To assess the impact of the uncertainty on the outcomes of the model, one-way sensitivity analyses were performed on the separate parameters and grouped parameters. Some parameters were grouped based on their impact on the development of CHIKV in humans and mosquitoes, the inclusion and effectiveness of countermeasures, vaccine efficacy, mosquito births and mortality, and the biting rate, see S5 Table.

In the sensitivity analyses each parameter was reduced and increased by 10%. The parameters in the formulae of the temperature-dependent lifecycle development and mortality were not varied in this analysis, however, these were assessed manually which resulted in extreme outcomes ranging from 0% to 100% of the population being infected. However, scenarios were assessed that evaluated the difference in the temperature used in the model. These scenarios included changing the method of temperature smoothing using the other polynomial functions investigated and including scenarios related to global warming. The impact of global warming could influence more than just the temperature, however in these scenarios the temperature for the entire years was shifted up by 0.25 degrees and 0.5 degrees Celsius.

## Results

This study models the effectiveness of emergency vaccination in controlling a hypothetical CHIKV outbreak in the city of Rome, Italy, considering different vaccination coverage scenarios. Using a dynamic disease transmission model, we simulate the spread of CHIKV within human and mosquito populations under varying environmental conditions and response strategies. Our model projects that an CHIKV outbreak in Rome could result in approximately 170,762 infections, accounting for 6.21% of the local population.

Introduction of an emergency vaccination program could substantially reduce the impact of the outbreak depending on the vaccine coverage rate. The number of cases decreases to 115,231 with a coverage of 10%; 76,031 with a coverage of 20%; 49,048 with a coverage of 30%; and 30,957 with a vaccine coverage rate of 40%. This represents a reduction of 55,531, 94,730, 121,714, and 139,805 cases for the coverage rates of 10%, 20%, 30%, and 40% respectively, thus preventing 33%, 55%, 71%, and 82% of the potential infections respectively. Notably, even with lower vaccination coverage rates, significant benefits are observed.

### Validation

The peak vector-host ratio that was estimated by the model calibrated to the Anzio outbreak was 7.2, which closely matched the peak vector host ratio reported for the Anzio outbreak of 7.3 [41]. This supports the calibration that was performed using the egg-carrying capacity as the size of the predicted and observed mosquito populations match. For the Rome outbreak the model estimates a vector-host ratio of 3.7. This is lower than the Anzio outbreak as expected, yet still within the range reported for this outbreak [41].

To identify potential outbreaks with similar vector-host ratios to the vector-host ratio estimated for the Rome outbreak, the outcomes of a systematic literature review were used. The aim was to find studies that reported both outbreak size using attack rates and vector-host ratios. The search was performed March 2023 using the search strategy and inclusion/exclusion criteria are reported in S1, S2, and S3 Tables. None of the identified studies that reported the attack rates also reported vector-host ratios, as is shown in S4 Table. Therefore, the estimated outbreak in Rome could not be validated using outbreaks with similar vector-host ratios.

In Europe, five outbreaks have occurred including the Anzio outbreak. Three outbreaks occurred in southern France and two have occurred in Italy [43]. Table 6 shows some of the key details of the European outbreaks. The Italian outbreaks are larger than the outbreaks that occurred in France, this could be due to the high degree of *Ae*. *Albopictus* infestation in Italy [12]. The outbreaks in Frejus and Montpellier occurred in a later period of the year and are therefore not comparable to the Anzio outbreak. The outbreak in Le-Cannet-des-Maures started at the end of July, was contained in a small area, and the strain originated in Africa instead of India [43]. This combined with the differences in infestation between France and Italy, leads to the exclusion of this outbreak for validation of the Rome outbreak. After the outbreak in Emilia Romagna a seroprevalence study was performed which found a prevalence rate of 10.2% (95% CI: 7.1–14), which is higher than the estimated outbreak size of 6.21% in Rome.

**Table 6:** Location, timing, and size of the European outbreaks.

Outbreak location	Year	Period	Number of cases
Italy, Emilia Romagna	2007	July-September	330
France, Fréjus	2010	September	2
France, Montpellier	2014	September -October	12
France, Le-Cannet-des-Maures	2017	July-September	17
Italy, Anzio	2017	June-November	489

The Anzio outbreak contained a focus in Rome which resulted in 80 cases [20]. The first cases in Rome presented with symptoms between 1 and 3 September [44]. Using this information, the Rome outbreak in the model was started on 2 September resulting in an outbreak of 82 cases. This outcome supports the validity of the model outcomes.

### Sensitivity and scenario analyses

The effectiveness of any vaccination programme depends on the coverage rate as more vaccinated individuals prevent the infection from spreading and further flatten the outbreak curve. No previous emergency vaccination programme for CHIKV has been implemented. Therefore, it is unknown how many people would want to take the vaccine. To estimate the impact of the different coverage rates on the number of infections in the Rome outbreak a series of scenario analyses have been performed. In these scenarios, the coverage rate was increased from 0% to 80% with ten percentage point intervals. The results from the scenario analyses are presented in S6 Table using a coverage rate of 40% to indicate the maximum impact of vaccination.

Additionally, scenario analyses were performed using different rates for vaccine effectiveness. Increasing the vaccine effectiveness from 98.9% to 100% would reduce the number of infections with vaccination from 30,957 to 30,312, representing 1.13% and 1.10% of the population respectively. A lower vaccine effectiveness of 90% would result in an outbreak with 36,653 cases and 1.33% of the population being infected.

In an additional sensitivity analysis parameters included in the model are varied separately. Each parameter included in the model was increased by 10% and decreased by 10% sequentially. For each change in the parameters, the percentage of individuals infected in the scenario where vaccination is implemented is recorded. The change in this outcome between the scenario and the base case measures the impact of parameter uncertainty. The parameters with the largest impact are the urban population density, the multiplier of adult mortality, the number of blood meals per gonotrophic cycle, and the human population density in Rome. While the base case resulted in 1.13% of the population infected, changing these parameters led to infection rates ranging from 0.22% to 5.33%. The outcomes of this sensitivity analysis are shown in a tornado diagram in S3 Fig.

The sensitivity analysis described above for the scenario where vaccination is implemented has also been performed for the scenario where vaccination is not implemented. The outcomes of the sensitivity analysis in the scenario without vaccination are shown in a tornado diagram in S4 Fig. The parameters with the largest impact are the same for the scenario with and the scenario without vaccination. The base case without vaccination resulted in 6.21% of the population infected, changing the parameters led to infection rates ranging from 0.86% to 36.71%. The largest impact of vaccination would thus reduce the infection rates from 36.71% to 5.33% of the population being infected.

The parameters that resulted in the largest impact on the number of infections in Rome were parameters related to the biting rate, development of CHIKV in mosquitoes, mosquito births, and mosquito survival. Each of these groups of parameters resulted in over 40% of the population being infected without vaccination when changed by 10%, see S6 Table.

Scenario analyses were conducted to assess various factors that influence the dynamics of a potential outbreak, including the timing of the virus introduction, adjustments in land area ratios when extrapolating from Anzio to Rome, the identification timing of the outbreak, and the effects of global warming. Detailed results of these analyses are presented in S6 Table.

Modifying the outbreak’s timing by increments of a week consistently demonstrated a reduction in the outbreak size, with the most significant decrease observed when the CHIKV introduction occurred one week earlier, resulting in infection rates of 5.48% without vaccination and 1.02% with vaccine intervention. The model adjustments for land area from Anzio to Rome considered proportional changes in the initial number of eggs and the egg-carrying capacity, applying a minor adjustment factor of 5%. Additional scenarios applied different rates of land area proportionality particularly 10% and 0%, leading to infection rates of 3.73% and 10.02%. The analysis also varied the number of diagnosed cases necessary to trigger outbreak identification from 0 to 400, affecting the subsequent outbreak sizes from 0.24% to 3.27% of the population under vaccination scenarios. This highlights the critical role of timely outbreak identification, where early detection after 30 cases could prevent up to 75% of potential cases, whereas identification after 300 cases reduced prevention effectiveness to 50%.

Additionally, the impact of timely response following outbreak detection was evaluated. The response time is determined by the time from outbreak detection to the rollout of the emergency vaccination and the duration of the rollout. In the base case the time until emergency vaccination is 14 days, and the duration of the rollout is 30 days. In the scenario analyses both times were reduced and expanded by 7 days to evaluate the impact of intervention timing. The time from outbreak identification to vaccination rollout of 7 days and 21 days resulted in infection rates of 0.89% and 1.42% of the population. The duration of vaccine rollout of 23 days and 37 days resulted in infection rates of 0.98% and 1.3% of the population.

Furthermore, scenarios incorporating global warming, with annual temperature increases of 0.25 and 0.5 degrees Celsius, projected substantial increases in the infection rates to 10.16% and 17.05% without vaccination, respectively. These results underscore the significant impact of environmental changes on outbreak potential, its link to climate change, and the need for effective intervention strategies.

Overall, the sensitivity and scenario analyses highlight the inherent uncertainty in the model. Parameter uncertainty is an important source of uncertainty due to the heterogeneity in reported outcomes in the literature which was also highlighted in meta-analyses of chikungunya [7–9]. Furthermore, there is large uncertainty surrounding the mosquito lifecycle-related parameters. These parameters have not been included in the sensitivity analyses as mentioned previously, changing these parameters would result in an outbreak size of 0% or 100% of the population depending on the changes in the values. The sensitivity analyses found four cases where changing the parameters resulted in an outbreak size smaller than 1% or larger than 10% which are the parameters related to the development of chikungunya in mosquitoes, mosquito births, mosquito survival, and the biting rate. Especially the biting rate has a large influence on the outcomes with the scenario analyses showing an outbreak size ranging from of 0.04% to 92.33%.

## Discussion

The expansion of the habitat of *Ae*. *albopictus* to more temperate regions increases the risk of outbreaks of arboviral disease outbreaks in Europe. With the ongoing adaptation of the species to colder climates and global warming, outbreaks are expected to occur more frequently and with greater intensity. With our model, we aimed to demonstrate the potential threat of a large outbreak of chikungunya in a major city in Europe, Rome, and to estimate the potential impact of emergency vaccination on the size of the outbreak. The annual risk of such as outbreak occurring could not be assessed and was deemed beyond the scope of the current work.

Our model showed that if in the hypothetical scenario the 2017 Anzio CHIKV outbreak would have originated in Rome, it would have led to 170,762 infections, corresponding to 6.21% of the city’s population. However, the implementation of an emergency vaccination program could substantially mitigate these effects, preventing the majority of infections (139,805) at a vaccine coverage rate of 40%. This demonstrates the profound impact of vaccination, even when initiated after the outbreak’s onset, highlighting its potential effectiveness as outbreak response measure.

The 2017 Anzio outbreak resulted in 0.68% of the population being infected. The hypothetical outbreak in Rome resulted in a higher percentage of infections, mainly as a result of the higher population density in Rome compared to Anzio. In the scenario and sensitivity analyses population density was found to be an influential parameter. The estimated number of infections during the outbreak originating in Rome is significantly greater than the 80 cases reported from the secondary foci in Rome, which stemmed from the 2017 Anzio outbreak. This discrepancy is attributed to the suboptimal timing and corresponding climate conditions that prevailed during the spread of the secondary foci, as demonstrated by the outbreak size estimated by the model when the timing of the outbreak was aligned with the start of the focus in Rome.

Additionally, our analysis suggests that even low levels of vaccine coverage can have significant benefits for outbreak control. Even with as low as 10% coverage, about one-third of potential infections could be prevented and over half of the infections would be prevented by reaching a coverage rate of 20% locally. This is particularly beneficial in the context of vaccine hesitancy post-COVID-19, suggesting that substantial benefits can be achieved even with minimal public compliance.

To mitigate the public health risks associated with arboviral diseases, authorities have two primary strategies: vector control to reduce the population of *Ae*. *albopictus* mosquitoes and immunization strategies to enhance population immunity. The predominant vector control method involves the use of insecticides. However, in Europe, the deployment of insecticides, particularly through aerial spraying and within sensitive areas such as public parks, sports grounds, and in the vicinity of hospitals and schools, is heavily regulated due to concerns over environmental and health impacts [45]. Moreover, the effectiveness of insecticides may diminish over time as mosquito populations develop resistance [46].

On the immunization front, the implementation of vaccination programs offers a proactive approach to disease control. Generating immunity within the community through vaccination can significantly reduce the incidence and severity of outbreaks. Localized and timely emergency vaccination interventions can be particularly effective in controlling outbreaks at their inception. For such strategies to be effective, it is crucial to maintain local stockpiles of vaccines and ensure the capacity for rapid outbreak detection and response.

Our study results were subject to several limitations. Firstly, modelling assumes homogenous mixing between the mosquito and the human population is not fully realistic as mosquitoes cannot travel across the entire metropolitan area of Rome. However, even though outbreaks are likely to be geographically concentrated, the identification of multiple foci in the Anzio outbreak indicates that, through human movement, an outbreak could potentially spread throughout the entire city of Rome. Homogeneous mixing is commonly used in modelling to simplify the complex interactions between host and vector populations, thereby streamlining the computational requirements and making the model more manageable for analytical and predictive purposes.

Furthermore, the assessment of population density’s influence on the proportion of mosquito bites targeting humans relied on a limited dataset. Only two data points were available, from which a linear relationship was extrapolated. Challenges were encountered in obtaining accurate population density Figs for areas with variable human presence, such as Sapienza University in Rome. This site, despite its low residential population, experiences high daily foot traffic. Consequently, an estimated population density of 2250 people per km^2^ was assumed for modelling purposes, based on observational estimates rather than official census data. This assumption was necessary to evaluate the impact of population density on mosquito biting behaviour, acknowledging that such an approximation introduces a degree of uncertainty into our model.

Finally, significant variability in the data concerning mosquito populations and Chikungunya virus (CHIKV) transmission rates contributed to considerable uncertainty in our modelling outcomes. This was highlighted in the sensitivity analysis, where modifications to parameters could lead to infection rates varying from under 1% to over 40% of the population. Additionally, the available data from the Anzio outbreak comprised only aggregate infection numbers rather than time-resolved data, which limited the precision of our model calibration. More granular data on infection rates would enhance model accuracy and reliability, thereby improving outbreak predictions and response strategies.

In conclusion, this study underscores the need for outbreak preparedness against CHIKV in major urban centres at risk for chikungunya transmission like Rome. Our findings demonstrate the potential effectiveness of a rapid vaccination response, significantly reducing outbreak severity when implemented timely. Effective outbreak management will require a multifaceted approach, combining robust surveillance systems, strategic vaccine stockpiles for rapid response, and public health initiatives to improve vaccine acceptance among the population. Additionally, considering the limitations associated with traditional vector control methods, innovative strategies that are both effective and environmentally sustainable will be critical for long-term management and prevention of arboviral diseases.

## Supporting information

S1 Table MEDLINE In-Process search strategy for the systematic literature review. (PDF)

S2 Table MEDLINE and Embase search strategy for the systematic literature review. (PDF)

S3 Table PICOS criteria for the inclusion and exclusion of studies. (PDF)

S4 Table Studies from the systematic literature review that reported on an outbreak and whether they mentioned a vector-host ratio. (PDF)

S5 Table Grouping of parameters for sensitivity analysis. (PDF)

S6 Table Results of grouped sensitivity analyses and scenario analyses. (PDF)

S1 Fig Raw temperature series and polynomial smoothing curves, Rome. (TIF)

S2 Fig Coverage curve scenario analysis showing the number of infections at various vaccination coverage rates. (TIF)

S3 Fig Tornado diagram showing the results of a one-way sensitivity analysis conducted for the scenario with vaccination. (TIF)

S4 Fig Tornado diagram showing the results of a one-way sensitivity analysis conducted for the scenario without vaccination. (TIF)
