# Supplementary material for: Strategic vaccination responses to Chikungunya outbreaks in Rome: Insights from a dynamic transmission model
Source: PLoS Negl Trop Dis. 2024 Dec 9;18(12):e0012713. doi: 10.1371/journal.pntd.0012713 (PMC11658691; doi:10.1371/journal.pntd.0012713)
Supplement: S1 Table — (PDF) [file pntd.0012713.s001.pdf]

S1_Table. MEDLINE In-Process search strategy for the systematic literature review

String number	Query	Hits
1	“Chikungunya fever”[Mesh] OR “chikungunya”[tiab]	7,009
2	“case reports”[pt] OR editorial[pt] OR letter[pt] OR comment[pt] OR “clinical trial, veterinary”[pt]	4,250,526
3	#1 NOT #2	6,282
