# Supplementary material for: Strategic vaccination responses to Chikungunya outbreaks in Rome: Insights from a dynamic transmission model
Source: PLoS Negl Trop Dis. 2024 Dec 9;18(12):e0012713. doi: 10.1371/journal.pntd.0012713 (PMC11658691; doi:10.1371/journal.pntd.0012713)
Supplement: S2 Table — (PDF) [file pntd.0012713.s002.pdf]

S2_Table. MEDLINE and Embase search strategy for the systematic literature review

String number	Query	Hits
1	‘chikungunya’/exp OR ‘chikungunya’:ab,ti	9,622
2	'case study'/it OR 'case report'/it OR 'abstract report'/it OR editorial/it OR ‘veterinary clinical trial’/it OR letter/it OR note/it	2,933,263
3	#1 NOT #2	8,815
