# Supplementary material for: Strategic vaccination responses to Chikungunya outbreaks in Rome: Insights from a dynamic transmission model
Source: PLoS Negl Trop Dis. 2024 Dec 9;18(12):e0012713. doi: 10.1371/journal.pntd.0012713 (PMC11658691; doi:10.1371/journal.pntd.0012713)
Supplement: S3 Table — (PDF) [file pntd.0012713.s003.pdf]

S3_Table. PICOS criteria for the inclusion and exclusion of studies

**Category**	**Inclusion criteria**	**Exclusion criteria**
Population (P)	Chikungunya patients[a]<br>Furthermore, studies assess a mixed population were included regardless of the percentage of the study population[b]	Health volunteers<br>Diseases other than chikungunya, Zika, and Dengue
Intervention (I)	Any or no intervention	None
Comparator (C)	Any	None
Outcomes (O)	Attack rates	Studies that do not report at least one of the outcomes of interest
Study Design (S)	RCTs – both parallel-group and crossover<br>(double-blind, single-blind, open-label)	*In vitro* studies<br>Preclinical studies
	Systematic reviews and meta-analyses of RCTs<br>Non-RCTs<br>Retrospective and prospective cohort studies<br>Case studies<br>Single-arm trials<br>Real-world evidence studies	Reviews, comments, letters, and editorials
Language	English	None
Time limit	No restriction	None
Country	No restriction	None

Abbreviations: RCT, randomized controlled trial; AE, adverse events; QoL, quality of life

[a] Population with only CHIKV or coinfected with Zika and/or dengue.

[b] Mixed populations containing a chikungunya subgroup will be included and flagged.
