# Supplementary material for: Strategic vaccination responses to Chikungunya outbreaks in Rome: Insights from a dynamic transmission model
Source: PLoS Negl Trop Dis. 2024 Dec 9;18(12):e0012713. doi: 10.1371/journal.pntd.0012713 (PMC11658691; doi:10.1371/journal.pntd.0012713)
Supplement: S4 Table — (PDF) [file pntd.0012713.s004.pdf]

S4_Table. Studies from the systematic literature review that reported on an outbreak and whether they mentioned a vector-host ratio

Author (year)	Country/region	Vector-host ratio
Sitepu (2020) (48)	Indonesia	Not reported
Simião (2019) (49)	Brazil	Not reported
Van Genderen (2016) (50)	Suriname	Not reported
Ramachandran (2012) (51)	India	Not reported
Qiaoli (2012) (52)	China	Not reported
Yoosuf (2009) (53)	Maldives	Not reported
Sissoko (2008) (54)	Mayotte	Not reported
Sergon (2008) (55)	Kenia	Not reported
Rezza (2007) (56)	Italy	Not reported
Kumar (2017) (57)	Caribbean	Not reported
Chopra (2012) (58)	India	Not reported
Ayu (2010) (59)	Malaysia	Not reported
Manimunda (2010) (60)	India	Not reported
Borgherini (2008) (61)	La Réunion	Not reported
Ball (2019) (62)	Haiti	Not reported
Pastula (2017) (63)	Micronesia	Not reported
Balmaseda (2016) (64)	Nicaragua	Not reported
Kaur (2008) (65)	India	Not reported
Oviedo-Pastrana (2017) (66)	Colombia	Not reported
Kosasih (2013) (67)	Indonesia	Not reported
Alayu (2021) (68)	Ethiopia	Not reported
Carrera (2017) (69)	Panama	Not reported
Carrillo-Hernandez (2018) (70)	Colombia	Not reported
Cunha (2017) (71)	Brazil	Not reported
Gérardin (2008) (72)	La Réunion	Not reported

Gordon (2018) (73)	Nicaragua	Not reported
Khatun (2015) (74)	Bangladesh	Not reported
