# Supplementary material for: Strategic vaccination responses to Chikungunya outbreaks in Rome: Insights from a dynamic transmission model
Source: PLoS Negl Trop Dis. 2024 Dec 9;18(12):e0012713. doi: 10.1371/journal.pntd.0012713 (PMC11658691; doi:10.1371/journal.pntd.0012713)
Supplement: S5 Table — (PDF) [file pntd.0012713.s005.pdf]

S5_Table. Grouping of Parameters for Sensitivity Analysis.

Parameter	Varied separately and/or grouped	Group
Human susceptibility	Both	CHIKV development in humans
Intrinsic incubation rate	Both	CHIKV development in humans
Recovery rate	Both	CHIKV development in humans
Mosquito susceptibility	Both	CHIKV development in mosquitoes
Extrinsic incubation rate	Both	CHIKV development in mosquitoes
Percentage of female mosquitoes	Both	CHIKV development in mosquitoes
Efficacy adulticides	Grouped only	Countermeasures efficacy
Effectiveness vaccine	Both	Vaccine efficacy
Vaccine coverage	Both	Vaccine efficacy
Eggs per cycle	Both	Mosquito births
Minimum temperature egg-laying	Both	Mosquito births
Carrying capacity	Both	Mosquito births
Multiplier larval and pupal mortality	Both	Mosquito mortality
Multiplier adult mortality	Both	Mosquito mortality
Minimum temperature biting	Both	Biting rate
Blood meals per gonotrophic cycle	Both	Biting rate
Definition rural population density	Both	Biting rate
Percentage human feeding in rural areas	Both	Biting rate
Definition urban population density	Both	Biting rate
Percentage human feeding in urban areas	Both	Biting rate
