# Supplementary material for: Strategic vaccination responses to Chikungunya outbreaks in Rome: Insights from a dynamic transmission model
Source: PLoS Negl Trop Dis. 2024 Dec 9;18(12):e0012713. doi: 10.1371/journal.pntd.0012713 (PMC11658691; doi:10.1371/journal.pntd.0012713)
Supplement: S6 Table — (PDF) [file pntd.0012713.s006.pdf]

S6_Table. Results of Grouped Sensitivity Analyses and Scenario Analyses.

**Scenario/grouped sensitivity**	**Percentage of the population infected – no vaccine**	**Percentage of the population infected – with vaccine**
Base case results	6.21%	1.13%
Number of cases needed for outbreak identification - 0	6.21%	0.24%
Number of cases needed for outbreak identification – 30	6.21%	1.61%
Number of cases needed for outbreak identification – 50	6.21%	1.88%
Number of cases needed for outbreak identification – 100	6.21%	2.31%
Number of cases needed for outbreak identification – 150	6.21%	2.59%
Number of cases needed for outbreak identification – 200	6.21%	2.81%
Number of cases needed for outbreak identification – 250	6.21%	2.96%
Number of cases needed for outbreak identification – 300	6.21%	3.12%
Number of cases needed for outbreak identification – 350	6.21%	3.19%
Number of cases needed for outbreak identification - 400	6.21%	3.27%
Land area proportionality – 90%	3.73%	0.73%
Land area proportionality – 100%	10.02%	1.71%
Date of arrival CHIKV index case – 04 June	6.20%	1.12%
Date of arrival CHIKV index case – 06 June	6.19%	1.12%
Date of arrival CHIKV index case – 29 May	5.48%	1.02%
Date of arrival CHIKV index case – 12 June	5.48%	1.02%
Temperature smoothing - 2nd order polynomial	1.57%	0.31%
Temperature smoothing - 4th order polynomial	6.67%	1.41%
Temperature smoothing - 5th order polynomial	6.90%	1.64%
Temperature smoothing - 6th order polynomial	6.31%	1.50%
Global warming - 0.25 degrees Celsius	10.16%	1.63%
Global warming - 0.5 degrees Celsius	17.05%	2.57%
Development of CHIKV in humans – outbreak increase	28.88%	4.63%
Development of CHIKV in humans – outbreak decrease	1.14%	0.27%

Development of CHIKV in mosquitoes – outbreak increase	51.37%	8.99%
Development of CHIKV in mosquitoes–outbreak decrease	0.57%	0.16%
Include countermeasures	3.24%	0.60%
Include countermeasure - reduced efficacy	3.25%	0.60%
Include countermeasures – improve efficacy	3.23%	0.60%
Vaccine efficacy – reduced efficacy	6.21%	1.55%
Vaccine efficacy – improved efficacy	6.21%	0.91%
Mosquito births - outbreak increase	44.55%	7.10%
Mosquito births - outbreak decrease	0.28%	0.11%
Mosquito survival - outbreak increase	43.58%	7.47%
Mosquito survival - outbreak decrease	0.74%	0.19%
Biting rate - outbreak increase	92.33%	32.68%
Biting rate - outbreak decrease	0.04%	0.02%
