# Supplementary figures and images for: Strategic vaccination responses to Chikungunya outbreaks in Rome: Insights from a dynamic transmission model
Source: PLoS Negl Trop Dis. 2024 Dec 9;18(12):e0012713. doi: 10.1371/journal.pntd.0012713 (PMC11658691; doi:10.1371/journal.pntd.0012713)

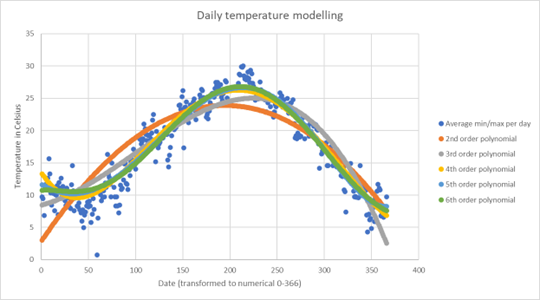

Supplement: S1 Fig — (TIF) [file pntd.0012713.s007.tif]

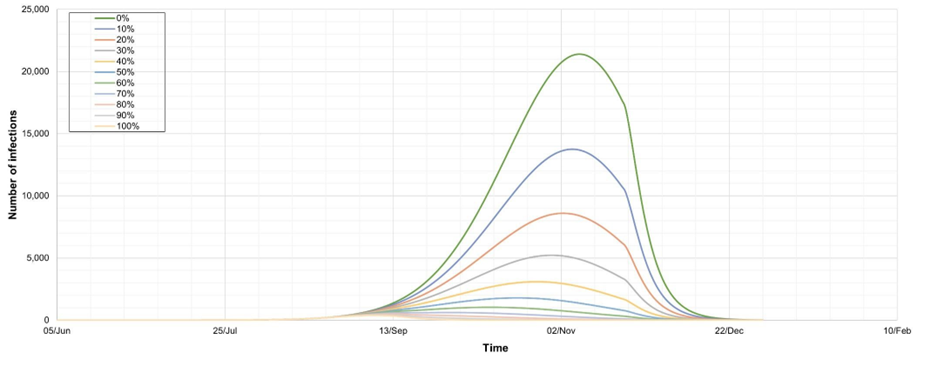

Supplement: S2 Fig — (TIF) [file pntd.0012713.s008.tif]

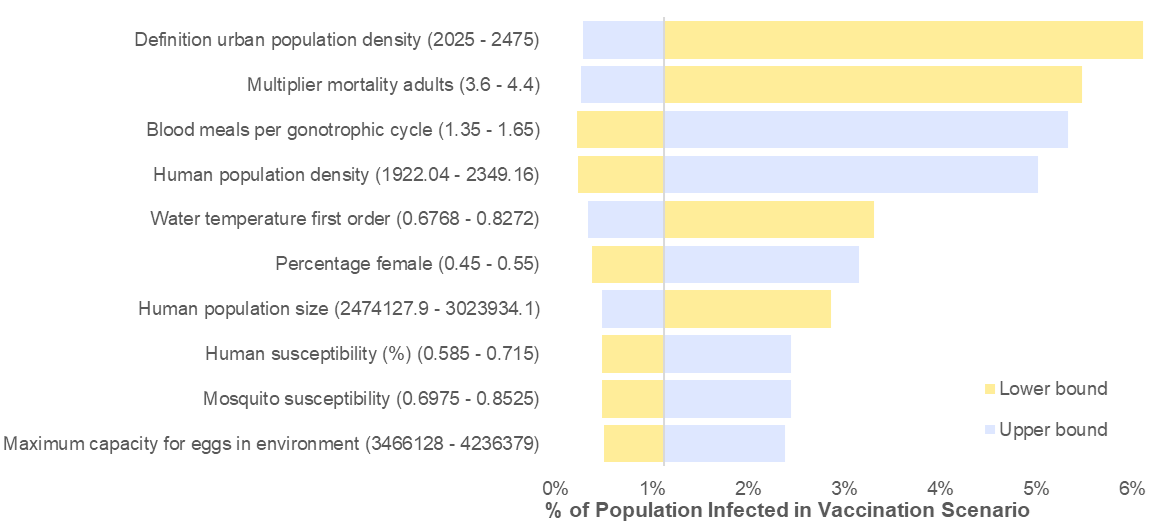

Supplement: S3 Fig — (TIF) [file pntd.0012713.s009.tif]

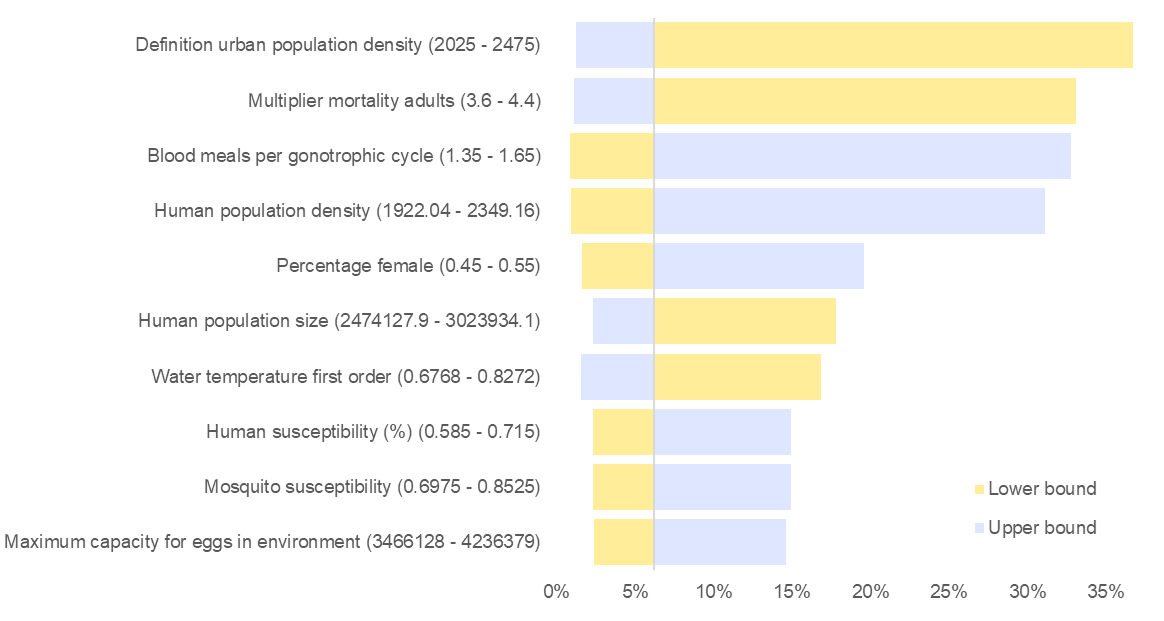

Supplement: S4 Fig — (TIF) [file pntd.0012713.s010.tif]
